# Global systematic mapping of *Vibrio* species pathogenicity: A PRISMA-guided cluster-based analysis

**DOI:** 10.1097/MD.0000000000041664

**Published:** 2025-02-28

**Authors:** Hope Onohuean, Uchechukwu U. Nwodo

**Affiliations:** a Biopharmaceutics Unit, Department of Pharmacology and Toxicology, School of Pharmacy, Kampala International University Western Campus, Ishaka-Bushenyi, Uganda; b Biomolecules, Metagenomics, Endocrine and Tropical Disease Research Group (BMETDREG), Kampala International University, Western Campus, Ishaka-Bushenyi, Uganda; c Patho-Biocatalysis Group, Department of Biochemistry and Microbiology, University of Fort Hare, Eastern Cape, South Africa.

**Keywords:** bibliographic-coupling-techniques, co-citation-analysis, pathogenesis, vibriosis, virulence toxins

## Abstract

**Background:**

A systematic global map on toxigenesis and genomic virulence of *Vibrio* spp. was analyzed for research progress to identify the emerging research patterns for establishing a database for designing future interventions.

**Method:**

The databases (Web of Science and Scopus) were analyzed with Voxviewer software to map the global scale of *Vibrio* spp. or virulence toxin/genes publications and standardized using Preferred Reporting Items for Systematic Reviews and Meta-Analyses (PRISMA) strategies.

**Results:**

The results identified 1162 (Web of Science n = 620, Scopus n = 542), while 314 studies qualified for inclusion and were significantly analyzed on virulence toxin/genes. By co-citation analysis, 4-thematic clusters were developed from 6420 citations and a total reference of 9062. Cluster #1 (pathogenesis & virulence factors) and cluster #4 (host response factors) generated the utmost publications and citations (n = 40, 643) and the least (n = 7, 85) interest by the researcher. Whereas 8-thematic clusters were developed by bibliographic coupling technique analysis, cluster#1 and cluster#8 generated the utmost (n = 78, 1684) and least (n = 7, 81) publications and citations interest by the researcher.

**Conclusions:**

Our findings suggest that *Vibrio* toxigenesis and virulence factors are a complex field requiring an interdisciplinary approach consisting of interconnected perspectives that have important consequences for academics and policymakers.

## Graphical abstract

**Figure:**
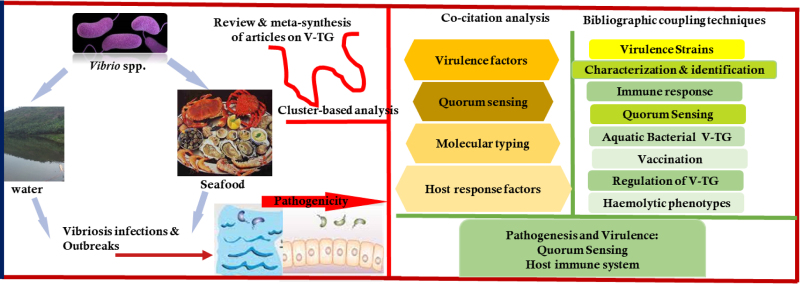

## 1. Introduction

*Vibrio* species (*Vibrio* spp.) are universally distributed worldwide in nautical and estuarine milieus.^[1,2]^ Most of the species are halophilic and entail 0.5 to 3% NaCl to thrive. *Vibrio* spp. is found in various aquatic, estuarine, and freshwater locale sediments and in coexistence with plankton and nautical organisms.^[3,4]^ Virulence influences of concern make *Vibrio* pathogens able to cause infection in the host. The pathogenesis of vibriosis includes adherence, entrance, toxigenesis, multiplication, defense system evasion, infections, and damage to the infected host.^[5]^ For *Vibrio* spp., virulence determinants include capsular polysaccharides, adhesive factors, cytotoxins, lipopolysaccharides, and flagellum.^[6]^ They are implicated in human pathogenic *Vibrio* spp. and aquatic vertebrates or invertebrates. *Vibrio* cholera and noncholera *Vibrio* infection, or the group causing gastrointestinal/extra-intestinal diseases, are the most prominent classifications of *Vibrio* infections. Among the over 100 identified vibrionaceae, 12 are human pathogens that cause diarrhea, septicemia, and extra-intestinal illnesses, like wound infections.^[1–4,7]^ After contact with these pathogens from contaminated water or food, attachment is an obligatory prerequisite to *Vibrio* colonization and infection, manifested in the case of gastrointestinal tract and wound infections.^[8–10]^

*V. cholerae* 01 highly virulent strains are aggressively motile and rapidly adhere to epithelial cell surfaces in the host, while isogenic mutants that are not motile/other mutagenized strains are attenuated.^[11]^ *V. parahaemolyticus* possesses a flagellum and hemagglutinins used in its adherence properties and cell surface factors,^[12]^ regulating resistance to naturally occurring host defenses and complement-mediated lysis. Capsule-like substance extant at the exterior of *V. vulnificus* strains enables resistance to lysis mediated by the complement of human serum and virulence in mice, fishes, and lobsters. Other attachment structures include adhesins,^[12]^ some of which mediate the agglutination of erythrocytes found in *Vibrio* spp. Several virulence factors, mutually cells-associated or extracellular proteins of manifold biological functions and properties, are implicated in developing *Vibrio* infections. Understanding the pathogenic factors, extracellular proteolytic toxins, surface cell association, and their interactions with other microorganism-produced compounds that aid virulence processes in the emerging and reemerging vibriosis is still a mirage. The emergence and reemergence of *Vibrio* infections are associated with variations of virulence toxins of the pathogenic species, a global health burden that requires attention. Using science mapping analytics to generate information from existing databases to identify research engagement will aid the design of effective *Vibrio* infection intervention. Furthermore, quantitative review techniques in virulence toxin/genes (V-TG) or *Vibrio* pathogenesis are still limited, particularly concerning capturing the advancements or research progress in the field (for calls to close this gap). This study reviews the global research on toxigenesis and genomics of virulence *Vibrio* spp., from a bibliometric standpoint, applying science mapping techniques. We examined the emergence of research in *Vibrio* species pathogenicity and identified the primary emerging research patterns with the ultimate goal of establishing a database for designing future interventions.

## 2. Methods

For this study, published research articles were salvaged from the Web of Science and Scopus databases following the “Preferred Reporting Items for Systematic Reviews and Meta-Analyses” (PRISMA) technique,^[13]^ for three decades of synthesis (1999–2020) adopting the method^[14]^ in data analysis as shown in Figures S1 and S2, Supplemental Digital Content, http://links.lww.com/MD/O432. Two authors separately performed a Boolean search for articles on V-TG using the keywords: (virulence toxins*) OR (virulence genes*) OR (virulence determinants) OR (virulence signatures) AND (Vibrio species*) OR (Vibrio spp. *) OR (V. Species*) OR (Vibrio pathogens*)” in the ISI Web of Science and Scopus databases by refining to the topic which covers the title of articles, their abstracts and keywords. All published articles used for bibliographic coupling analysis based on the set of references used for co-citation analysis were examined for the presence of the ‘V-TG’ keyword in the title and abstract to eliminate untrue positive items in the metadata (Figures S1 and S3, Supplemental Digital Content, http://links.lww.com/MD/O432). Only articles that mention/have the virulence genes, virulence determinant, virulence signature, and *Vibrio* spp. or any of the *Vibrio* species. Studies of which V-TG were identified by conventional phenotypic or genotypic methods, whole genome sequencing or polymerase chain reaction, and matrix-assisted laser desorption ionization–time of flight mass spectrometry were qualified for inclusion.

**Figure 1.**
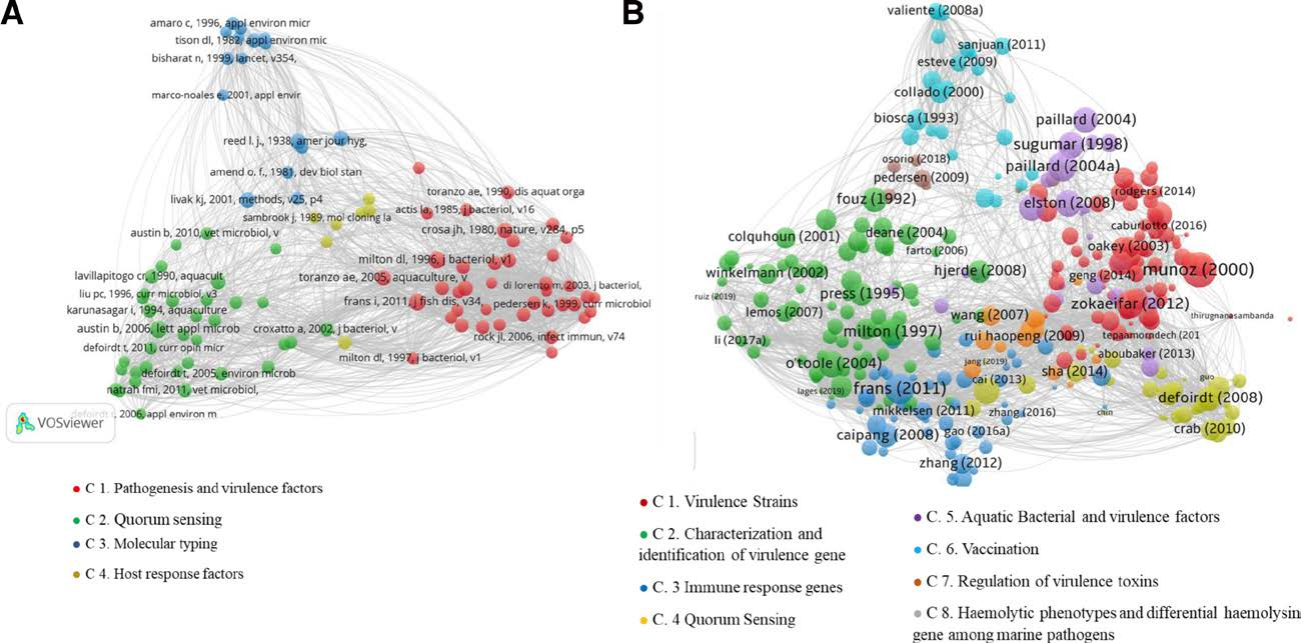
(A) Cluster-based visualization network of co-cited articles included in the dataset. (B) Cluster-based visualization network of shared reference articles included in the dataset.

The study evaluated and mapped the similarity of V-TG research themes using co-citation analysis and bibliographic coupling techniques according to Boyack and Klavans^[15]^ in Figures 1A, B. Imperatively, co-citation analysis focuses on matching published articles in the dataset that are jointly or co-cited by another article, whereas bibliographic coupling focuses on matching published articles in the dataset based on shared references. However, co-citation analysis is primarily based on cited articles, making it appropriate for examining the evolution of literature roots, and bibliographic coupling techniques are primarily based on citing articles, making them more applicable to identifying the current state of the literature and emerging patterns.^[16]^

We analyzed the retrieved dataset with visualization of similarities (VOS)viewer 1.6.13 version as follows:

1. The thematic cluster was developed and visualized based on the strength of association and relatedness^[17,18]^ of co-occurrence data.
2. The association strength was calculated and compared within the dataset.^[17]^
3. The dataset cluster networks were created using the VOS algorithm.^[18]^

### 2.1. Ethical approval

This study did not require ethical approval as the scientific evidence contained in this study was meta-synthesized from already published available articles.

## 3. Results

Figure S2, Supplemental Digital Content, http://links.lww.com/MD/O432 shows the search terms, the procedure details for retrieving the result document type of articles, and literature published on V-TG within the period of 1990–2019. A total of 314 full articles were published with 6420 citations, and a total reference of 9062 were obtained.

### 3.1. Co-occurrence of all keyword analysis results

The searched keywords’ co-occurrence mapping analysis by the counting method reference is presented in Figure S3, Supplemental Digital Content, http://links.lww.com/MD/O432. Analyzing all keywords’ co-occurrence mapping analytics of full counting showed 1784 keywords having the top 146 thresholds of a minimum occurrence.

### 3.2. Co-citation results

In Figure 1A, 4 clusters were labeled based on the cited references from the 314 published articles. However, a qualified dataset of 314 articles published between the study span of 1991–2019 depicts 6420 citations and 9062 references, which were standardized to the same format for further analysis. Therefore, to avoid overlapping complications and straightforward interpretation^[16]^ of essential documents, the top 10 publications of the most cited co-citation analysis were included, refining to 98 references that meet the threshold. All cited literature before 1990 was included to appraise the evolution of “virulence toxins/genes” in vibriosis investigation, and the quantity of references was utilized in co-citation analysis.

**Figure 2.**
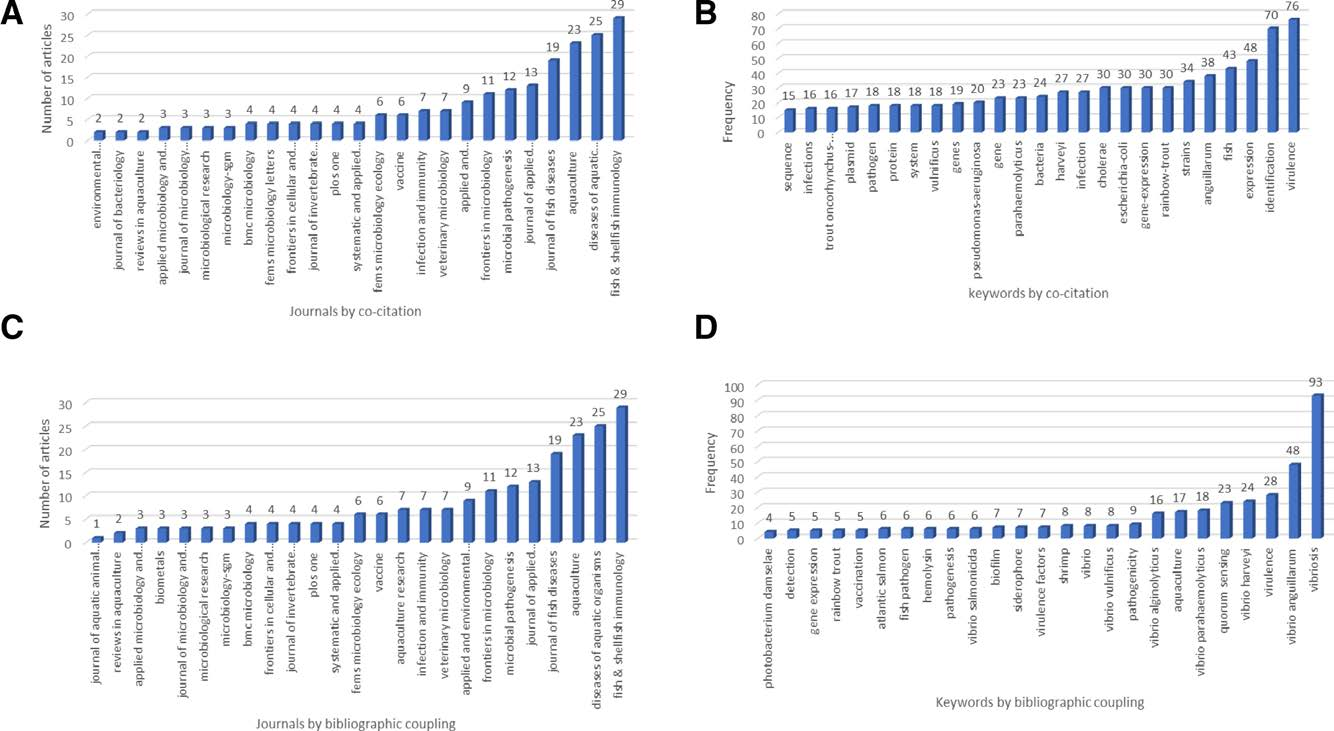
(A) Twenty-five most popular journals, based on the number of scientific publications by co-citation analysis. (B) Twenty-five most frequent keywords assigned to the articles, by co-citation analysis. (C) Twenty-five most popular journals, based on the number of scientific publications by bibliographic coupling technique. (D) Twenty-five most frequent keywords assigned to the articles, by bibliographic coupling technique.

The network visualization shows each scientific publication with a distinctive cited reference, which is clustered based on the probability of being cited with other articles. Each cluster has items likely to be cited together in another article. Colors denote clusters as well as the publications that belong to them. Furthermore, each item is given a specific weight based on the publication’s total link strength and the amount of received citations. Articles with a greater total link strength are visualized in larger-size nodes.^[18]^ Using the VOS approach and the co-citation analysis technique, the current trend of (V-TG) research was divided into 4 thematic clusters and labeled based on the authors’ independent content analysis, as depicted in Table 1. Furthermore, while we defined the clusters to reflect the entire V-TG literature, the foci of the perused articles were broad and representative. Information recovered was used to examine the progression of journal publication on V-TG and the associated keywords in the analyzed publications. The result is presented in Figure 2A. Among the top 25 leading peer-reviewed journals on V-TG in the study timespan, Fish and Shellfish Immunology published the highest number of articles, with 29 publications. The Diseases of Aquatic Organisms took the second leading position by publishing 25 articles, followed by the journal Aquaculture publishing 23 articles. Afterward, the prominent keywords in the cited articles were evaluated, and the rate of occurrence was recorded (Fig. 2B).

**Table 1:** Array of publications by co-citation

Clusters	Number of publications	Total citations per cluster	Total link strength	Average existence of publications (years)
1. Pathogenesis and virulence factors	40	643	6608	1.18
2. Quorum sensing	35	481	3724	1.21
3. Molecular typing	16	241	1377	0.21
4. Host response factors	7	85	645	0.11

Source: Author’s own compilation on evaluation of co-citation analysis technique.

#### 3.2.1. Cluster 1: pathogenesis and virulence factors

The publications in this cluster were observed to have a common research theme centered on pathogenesis, focusing on understanding the impact of virulence factors expressed by the pathogens. They used one of the pathogenic species (*Vibrio anguillarum*) as a model in this cluster. *V. anguillarum* has been described to be among the causal agents of hemorrhagic septicemic disease,^[19]^ affecting over 50 fresh/saltwater fish species of distinct nautical, fresh/brackish water fish, bivalves, and crustaceans. Its high morbidity and mortality rates accumulate to economic loss and treated to aquacultural industries. The cluster could identify virulence influences consisting of chemotaxis and motility, an iron uptake system, lipopolysaccharides (LPSs), and extracellular products with proteolytic or hemolytic activity to impact the pathogenesis of vibriosis significantly. Hemolytic activity is the main suggested virulence factor responsible for hemorrhagic septicemia and diarrhea infections^[20]^ of *Vibrio* spp., while zinc metalloprotease^[21]^ is involved in the invasive mechanism aided by active motility invasion. Further research examines the virulence role of the flagellin gene of *V*. *anguillarum* by cloning, sequencing, and mutagenizing from pathogenic fish. DNA sequence found the *N* terminal deletion, double deletion, and 942-bp deletion enhanced the 50% lethal dose via immersion infection by 70- to 700-fold virulence^[21]^ in fish. Other research attributes the important essential element iron to play a pivotal role in the bacterial ability to thrive in the bodily fluids and tissues of the vertebrate host.

#### 3.2.2. Cluster 2: quorum sensing

This cluster centered on the influence of mutations in the quorum sensing (QS) system of *V*. *anguillarum* and *V. harveyi* on their virulence. Although the pathogenicity mechanisms are imprecisely understood, the research in this cluster revolves around the ability of *V*. *harveyi* toxins to attach and form biofilms, QS, to produce various extracellular products, including proteases and hemolysins, LPS, and the interaction with bacteriophage and bacteriocin-like substances.^[22]^ Bacterial cell-to-host communication, that is, QS, cumulates to the production of virulence factors such as caseinase, gelatinase, lipase, hemolysin, and phospholipase. The cluster also reveals the use of enzymatic plate assays to study QS activity expressed by *V*. *harveyi,* and the result implies that disrupting QS could be a viable alternative technique for combating *V*. *harveyi* infections. Meanwhile, cloning a transcriptional activator gene from pathogenic *Vibrio* spp. may be positive regulators of serine, metalloproteases, pigment, and biofilm production.

#### 3.2.3. Cluster 3: molecular typing

This cluster deals with molecular typing as an important infection control and monitoring of the prevalence of *Vibrio vulnificus* strains. Here, the literature reports studies on environmental isolates by assessing the bacteria’s phenotypic characteristics and modes of transmission. Clonal isolates were studied by biotyping, pulsed-field gel electrophoresis, and restriction fragment length polymorphism analysis of a polymerase chain reaction fragment; thus, this is needed for clinical samples and other species. However, studies to further elucidate the ecology of this emerging pathogen are lacking.

#### 3.2.4. Cluster 4: host response factors

This cluster focuses on research on signaling host response factors to pathogenic *Vibrio* species. Although it is the smallest (7 articles) thematic cluster in co-citation analysis, signaling and regulatory responses of virulence toxins depend on the type of association the *Vibrio* strains have with an animal host or aquatic host. Understanding the host response factors may also reflect the varied protagonists of *Vibrios* in organizing and sustaining microinches within the aquatic environment. Additionally, the literature in this cluster reports that human pathogenic *Vibrio* spp. expresses proteolytic enzymes that directly digest host proteins or are ramblingly involved in producing toxic protein factors during their pathogenicity.^[23]^ A few articles in the same cluster suggest a notable research development that revolves around the diversity/complexity of vibriosis host response factors, intraspecies activities, niche habitation, and possible evolution.

### 3.3. Bibliographic coupling results

The results obtained by bibliographic coupling techniques on V-TG subjects during the study timespan shown in Figure 1B define the future research perspectives. The same approaches used in co-citation analyses were adopted to assess bibliographic coupling results. In the bibliographic coupling analysis, no restrictions were applied to the number of citations perused to develop new insights based on previous studies. The 314 published articles were analyzed, while only 310 published articles had the largest connected items clustered and contained within the bibliographic network. The 4 published articles that were excluded had no communal references among other published articles in the obtained metadata and could not be interlinked. The retrieved information examines the progression and distribution of journal publications on V-TG and the associated keywords in the analyzed publications shown in Figure 2C. The top 25 leading peer-reviewed journals on V-TG by bibliographic coupling analysis during the study timespan showed that the first was Fish and Shellfish Immunology, with 29 publications, seconded by the Journal of Diseases of Aquatic Organisms with 25 publications, while the Journal of Aquaculture took third position with 23 articles, respectively. Afterward, the most frequent keywords were evaluated, and the rate of occurrence was recorded (Fig. 2D).

In addition, using the VOS approach and the bibliographic coupling technique, the research streams were divided into 8 thematic clusters and labeled based on the authors’ independent content analysis indicated in Table 2. Also, the clusters were defined to reflect the entire V-TG literature, and the foci of the perused articles were broad and representative.

**Table 2:** Array of publications, based on coupling references

Clusters	Number of publications	Total citations per cluster	Total link strength	Average existence of publications (in years)
1. Virulence strains mediated by experimental bacteriophage	78	1684	8660	2.71
2. Characterization and identification virulence gene	74	1576	14256	2.64
3. Immune response	50	776	6092	1.79
4. Quorum sensing	38	718	4700	1.29
5. Aquatic bacterial and virulence factors	26	860	2356	0.93
6. Vaccination	24	450	3681	0.86
7. Regulation of virulence toxins	13	248	2005	0.46
8. Haemolytic phenotypes and differential hemolysin gene among marine pathogens	7	81	656	0.25

Source: Author’s own compilation and evaluation from bibliographic coupling analysis technique.

#### 3.3.1. Cluster 1. Virulence strains

This is the first cluster with the largest articles; the research theme centered on virulence strains mediated by bacteriophages.^[24,25]^ To date, there is no clear clarification as to why some *Vibrio* strains are pathogenic while others are not, and most *Vibrio* strains act as opportunistic agents^[26]^ in secondary infections while others are recognized as pathogenic to humans and aquaculture. Several articles in this cluster demonstrate that experimental bacteriophages may confer virulence to the *V*. *harveyi* strain.^[26]^ Additionally, previous research in the cluster looked at the impact of probiotic *Bacillus* subtilis strains proceeding *Vibrio* spp., growth, enzymatic events, immune genes expressed, and resistance.

#### 3.3.2. Cluster 2. Characterization and identification of virulence gene

The second trendy cluster involves articles that focus on virulence gene identification and characterization, specifically hemolytic toxins as the core virulence influence involved in hemorrhagic septicemia and diarrhea of *Vibrio* infections.^[26]^ A fraction of articles in this cluster identified, characterized, and examined the role of hemolysin genes/hemolytic activity in virulence. Other groups of articles report the use of insertion sequence (IS) elements (gene acquisition)/small, freely mobile genetic elements as significant evolutionary event.^[24]^ The IS acts as a transposable element that catalyzes the intra-genome or inter-genome mobility in *Vibrio* virulence. Gene loss and chromosomal rearrangements^[24]^ are implicated in the uptake of iron, and the release of proteins with potential virulence role in *Vibrio* pathogenicity was also highlighted in this cluster. Later articles describe the identification and characterization of a QS circuit in *V. anguillarum* consisting of the LuxI homolog VanI, the LuxR homolog VanR, and the autoinducer molecule *N*-(3-oxodecanoyl)-L-homoserine lactone.

#### 3.3.3. Cluster 3. Immune response genes

This cluster of articles focuses on the immune system interaction during vaccination. Here, research attentions were on apolipoprotein A-I and nonspecific cytotoxic cell receptor protein,^[27]^ toll-like receptors, cytoplasm of NOD-like receptor C4,^[28]^ examples of cytotoxic-related and cell-mediated immunity genes is upregulation in the expression of tumor necrosis factor-α, and interleukins (IL) pro-inflammatory cytokines, IL-1, IL-1β, and IL-8^[23,29]^ following the intraperitoneal vaccination with heat-killed *Vibrio* spp., (Listonella/*Vibrio* anguillarum). The encoded apolipoprotein A-I is a 28.1 kDa protein composed of 243 amino acids and 21 peptides. It is situated on chromosome 11, with its specific location being 11q23-q24, with a gene containing 4 exons. Antibacterial genes, bactericidal/permeability-increasing protein/LPS-binding protein, g-type lysozyme, and transferrin expression were considerably elevated in the vaccinated mouse, according to the studies in this cluster.

Additionally, *Vibrio* infection begins once the toxins attach to mucosal surfaces like the stomach, animal skin, and fish gills. Some elements of this cluster investigate the impact of various mucosal components in fish upon *Vibrio* infection by mass spectrometry with two-dimensional gel electrophoresis. The steam articles,^[27–29]^ highlighted the key immune-relevant proteins, including small calpain subunit 1, glutathione-S-transferase omega 1, proteasome 26S subunit, 14-kDa apolipoprotein, beta 2-tubulin, cold inducible RNA binding protein, malate dehydrogenase 2 (mitochondrial), and type II keratin, which were found to have significant differential expression in cod skin mucus after natural infection with V. *anguillarum*. The cluster also has streams of literature that deal with the protective immunological mechanisms of live attenuated V. *anguillarum* in the zebrafish model. The result indicates an increase in the specific antibody response with a remarkable relative protection survival of about 90%, and expression of pro-inflammatory factors like IL-1 and IL-8 is increased upregulated.

#### 3.3.4. Cluster 4. Quorum sensing

The focus of this cluster is majorly on QS cascade events in regulating *Vibrio* virulence factors. QS signal molecules ([*N*-acyl homoserine lactones] [AHLs]), responsible for bacteria cell-to-cell communication, are found in some Gram-negative pathogenic bacteria that regulate virulence factors. The first thematic research in this cluster was the effect of inhibition of bacterial QS production to reduce mortality by *Vibrio* spp., further elucidating the mechanism of disease control of QS inhibitors (QSIs), furanone C-30, by determining its effect on the bacterial proteome, motility, and respiration. Second, thematic research in this cluster was the prevention of QS-mediated biofilm development and virulence factor production in *Vibrio* spp. Since the pathogenicity of vibriosis is controlled by the (QS) system, interfering with this machinery would prevent the pathogenicity of *Vibrio* without developing resistance. The last thematic articles investigate the regulation of QS specifically expressed by caseinase, gelatinase, lipase, hemolysin, or phospholipases. The cluster suggests that interrupting and regulating QS events negatively regulates phospholipase activity, as seen in mutants with low QS activity.

#### 3.3.5. Cluster 5. Aquatic, bacterial, and virulence factors

This cluster takes a dynamic view of the novel possible bivalve infections’ taxonomy and phylogeny, as well as their virulence factor associated with aquatic bodies. The cluster recognized vibriosis by the bacterial profile of the strains found in molluscan shellfish diseases. In addition, the bacterial silhouette revealed that *Vibrio* spp., established approximately 60 to 95% morbidity and mortality in marine organisms, *V*. *splendidus* biovar II and *V*. *harveyi*, were identified from the recovered isolates, suggesting their possible role in the disease. Furthermore, reemergence environmental drivers, such as ocean temperature elevation, were linked to dominant shellfish *Vibrio* pathogenicity, which upregulate the possession of genes coding for a protease and hemolysin found in *V*. *tubiashii*, pathogenic isolates that secreted peptides. However, the environmental factors influencing the mechanisms of host-pathogen interactions were highlighted. It suggests that a rise in temperature above 21°C may have a preventative effect, secretion of adherence, and cytotoxic factors in developing *Vibrio* infections.

#### 3.3.6. Cluster 6. Vaccination

Articles in this cluster report studies on evaluating various vaccine formulations against *V. vulnificus* serovar E (biotype 2)-caused vibriosis in an eels’ model. The studies in this theme explored attenuated live vaccines, inactivated entire cells with and without toxoids, inactivated extracellular products from capsulated and uncapsulated strains, and isolated LPS on eels model maintained in a controlled laboratory setting with a variety of delivery routes (injection and immersion). The types of the vaccine include whole-cell bacterin (WCB), toxoid-enriched bacterin (TWCB), virulence-attenuated live cells vaccine, and LPS-based vaccine.^[30]^

#### 3.3.7. Cluster 7. Regulation of virulence toxins

This cluster investigates the role of regulating functional genes that regulate the secretion of virulence toxins (extracellular protease, hemolytic materials, siderophore production, exotoxin, alkaline serine protease, flagellar proteins, as well as proteins involved in polysaccharide biosynthesis and transport). Although the cluster has a few articles because of the involvement of advanced laboratories, technology, and skills to design models in these new themes. However, articles in this cluster explore the regulations of gene expression and secretion, mutant deletions by extracellular autoinducer of protease activity, or extracellular products’ cytotoxicity that revolve around manipulating the expression of various virulence-related genes.

#### 3.3.8. Cluster 8. Hemolytic phenotypes and differential hemolysin genes among marine pathogens

Research in this cluster explores the diverse phenotype of hemolysin genes of the *Vibrio* damsela to understand hemolysin involvement in virulence. *Vibrio* damsela was first named and renamed Listonella damsela, later named Photobacterium damsela by Smith and his team,^[31]^ and subsequently validated as P. damselae subsp. damselae. The organism is reported as the main basis of mortality in turbot and several fish species^[32]^ in the Mediterranean. It was implicated to be pathogenic to mammals, including humans. However, little is known about this organism/bacterium globally. The stream of this cluster revealed that despite bacteria positive to virulence indicators such as hemolysis, motility, or urease, there is a substantial difference in their hemolytic characteristics. Furthermore, the cluster suggests that *Vibrio* coexists with other marine bacteria strains such as *Mytilus galloprovincialis,* the Pacific oyster *Crassostrea gigas*, *Pseudoalteromonas phenolica*, and *Aeromonas* sp. that yield the sturdiest hemolytic properties causing the pathological alterations seen in septicemia and hemorrhage. Thus, the diversity of hemolytic phenotypes frolicked and the distribution of plasmid-encoded hemolysins, is driven by horizontal gene transfer, gene duplication, and genetic variation. A foremost part in the formation of hemolysin gene gear in the pathogens.

## 4. Discussion

In this analysis, the enormous contribution of V-TG articles was elucidated to form a variety of research streams. However, the growing attention on this subject necessitates the synthesis of interdisciplinary biological insights into bacterial pathogenesis. Specifically, the results obtained from the bibliographic coupling technique and co-citation analysis of V-TG articles recommend numerous development axes that could benefit expanding V-TG research. The obtainable findings and research prospects are vital to the emergence and reemergence of pathogenic Vibrio infection in humans and aquaculture, which may influence researchers and policymakers.

**Figure 3.**
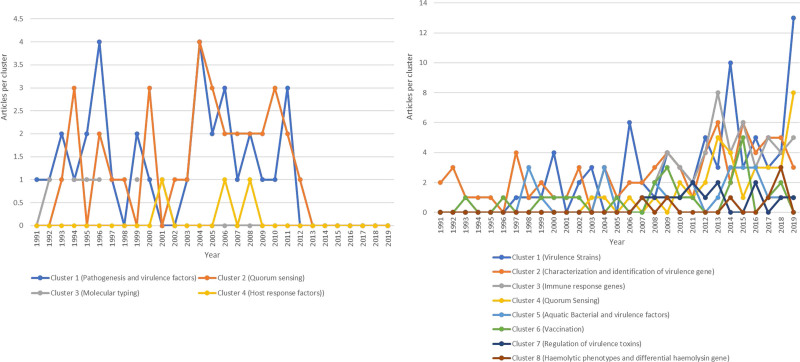
(A) The 4 evolving thematic clusters by co-citation technique. (B) The 8 evolving thematic clusters by bibliographic coupling technique.

V-TG research literature was expanded in the first two decades and became stagnant in the later half decade, perhaps due to better knowledge of *Vibrio* virulence using molecular diagnostic tools. According to research, the complexity of these toxin interactions is not well understood. Moreover, these species of *Vibrio* genomes express diversity in their virulence factors, while some lack pathogenic toxins. The main perceptions that offer the basis for V-TG research based on the co-citation analysis is presented in Figure 3A, thereby identifying 4 thematic clusters; cluster 1: pathogenesis and virulence factors, cluster 2: QS, cluster 3: molecular typing, and cluster 4: host response factors scopes of V-TG.

Using the bibliographic coupling technique to track the evolution of research literature on V-TG, thereby examining the transformation of the field’s present research dimensions as shown in Figure 3B. The virulence strains mediated by the experimental bacteriophage (cluster 1), QS (cluster 4), and immune response genes (cluster 3) shared a mounting research development and retained a position of importance as major topics in the studied period. The immune response (cluster 3) demonstrates an increased peak of research articles in 2008 and 2015, with slight maintenance to 2019, highlighting various factors influential to virulence toxins and the interaction process concerning *Vibrio* pathogenesis from other perspectives. The hemolytic phenotypes and differential hemolysin gene among marine pathogens (cluster 8) remained relatively stable with a major peak, suggesting that the research theme is not entirely explored and could be optimized. While the vaccination against vibriosis (cluster 6) and aquatic bacteria (cluster 5) has fluctuated with a major peak increase in the years 2008 to 2009 and 2015, 2013 to 2016, respectively, implying a research stream can be tapped into. The most protuberant suggestions for future investigation are related to the thematic in (cluster 7), regulation of virulence toxins, and (cluster 4) QS. In these research domains, new substreams that provide incentives for future engagement were discovered by the researchers. The quantitative bibliometric analysis of V-TG literature within the study timespan reveals notable inter-linkages of clusters among the main elements of V-TG research articles, suggesting a multi-layered research approach in this field. Precisely, the outcomes of the bibliographic coupling and co-citation techniques designate that research on V-TG can be synthesized into 4 interconnected thematics that may determine the leading research directions for the future built on the recognized clusters: pathogenesis and virulence (encompasses characterization and identification of virulence gene and virulence strains); QS: which corresponds to QS and aquatic bacterial virulence factors; host immune system: covering the immune response genes and vaccination against vibriosis strategy; regulation of toxin functional genes reflects the regulation of virulence toxins, pathogenesis, and hemolytic phenotypes, with differential hemolysin genes among marine/aquatic pathogens. The 4 interconnected streams/thematic address the possible mechanism of pathogenesis of vibriosis characterized by virulence factors. However, we acknowledge that most articles reported a poor/unclear understanding of the virulence toxins expressed by *Vibrio* spp., and most species have no tense literature on their virulences.

### 4.1. Pathogenesis and virulence

**Figure 4.**
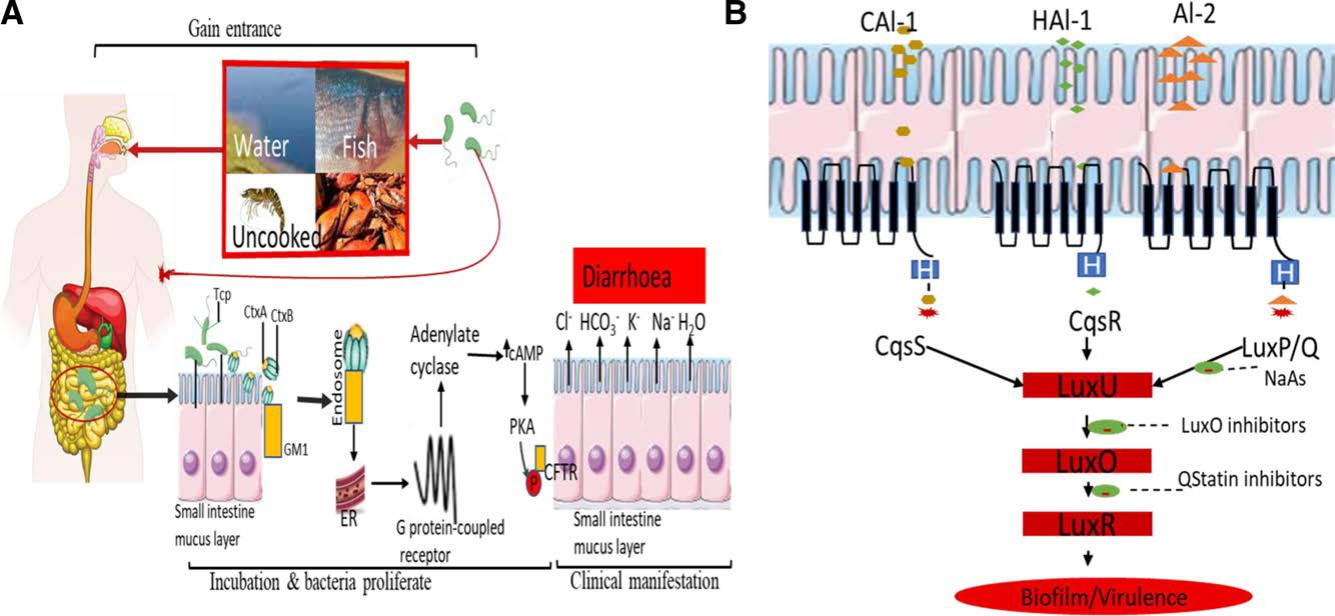
(A) Schematic representation on the mode of Vibrio spp., toxins entry & regulation inside the host. (B) Schematic relationship of QS & QSI & in Vibrio spp., pathogenicity.

Based on pathogenesis and virulence, the *Vibrio* genus has over 100 members consisting of 8 genera in the vibrionaceae order, namely, *Vibrio* sp., the *Aliivibrio*, *Catenococcus*, *Enterovibrio*, *Grimontia*, *Listonella*, *Photobacterium*, and *Salinivibrio* Bergey’s Manual of Systematic Bacteriology.^[33]^ Fourteen distinct clades have been revealed throughout evolutionary history including; *Photobacterium* clade, *Salinivibrio* clade, *Splendidus* clade, *Nereis* clade, *Orientalis* clade, *Coraliilyticus* clade, *Scophthalmi* clade, *Diazotrophicus* clade, *Cholerae* clade, *Anguillarum* clade, *Vulnificus* clade, *Halioticoli* clade, *Fischeri* clade, and *Harveyi* clade or *Vibrio* group.^[33]^ The clade genus shares both pathogenic and nonpathogenic bacteria. V-TG researchers recognize that vibrios belonging to the pathogenic clade show a high diversity of virulence factors, and these virulent members cause infections (vibriosis) and outbreaks with high mortality to both humans and the aquaculture sector, resulting in economic loss. The pathogenesis of *Vibrio* has been stated to transpire in 3 steps (Fig. 4A).

1. First, these pathogens gain entrance and attach themselves to the host from contaminated water, food/seafood, or open wounds, referred to as adhesion and/or penetration.
2. Second, a window or incubation time during which bacteria proliferate within the host with or without producing any obvious symptoms.
3. Third, the later phase of the disease’s clinical manifestation is convoyed by acute mortality. At this stage, there is a high level of pathogens in the host and the progression of infection to several tissues.

Given these characteristics, it is crucial to know which gene products are responsible for the bacteria’s pathogenicity as well as their expressions and distributions. Although the mechanism of *Vibrios* pathogenesis is not fully understood, some virulence-related factors and genes identified as responsible for various infections are discussed in Table S1, Supplemental Digital Content, http://links.lww.com/MD/O432. One of the well-known *Vibrio* spp., infections of economic significance and public health concern is cholerae. V. cholerae expresses a virulence factor called the toxin-co-regulated pilus and cholera toxin. Cholera toxin or choleragen (CTX, Ctx, or CT) is an AB5 multimeric protein complex. The toxin is a hexamer consisting of the CtxA subunit (part A, enzymatic, P01555) and the CtxB subunit (part B, receptor binding, P01556). Subunit B binds while subunit A activates the G protein, activating adenylate cyclase. The pentameric CtxB subunit works with CtxA and CtxB to bind to the ganglioside (a sialylated glycosphingolipid) GM1 on the plasma membrane of enterocytes. Cholera toxin that has been bound is endocytosed and is transported retrogradely to the endoplasmic reticulum (ER), where the subunits separate. The allosteric activation of the enzyme CtxA by adiponectin ribosylation factor 6 (ARF6) is made possible by releasing the CtxA subunit from the ER into the cytoplasm. The ARF6-bound, active CtxA subunit then catalyzes the adiponectin ribosylation of a G protein-coupled receptor, activating adenylyl cyclase. The cystic fibrosis transmembrane receptor is phosphorylated by protein kinase A in response to elevated levels of cAMP, which triggers the diarrhea outflow of ions and water into the small intestine (Fig. 4A). V-TG literature has captured research involving *Vibrio* pathogenesis associated with virulence factors, the proximity of *Vibrio* spp., opportunistic infections, or nonopportunistic and pathogenic or nonpathogenic strains remain challenging. Therefore, our findings urged research tailored to developing alternative rapid indicators to reliably determine both pathogenic and nonpathogenic *Vibrio* spp.

Additionally, V-TG research focusing on virulence mediated by experimental bacteriophages explores the existence of the bacteriophage *V. harveyi* myovirus-like, which may confer virulence to *V. harveyi* strains. In this context, the authors investigated the structural elucidation and identification of genes and putative genes for lysogeny by the complete nucleotide sequence of *V. harveyi* myovirus-like.^[25]^ The rise of concerns about the dissemination and the environmental propagation of antibiotic-resistant microorganisms requires promising alternative therapy, hence the use of lytic bacteriophages as therapeutic agents against bacterial diseases and to prevent vibriosis in aquaculture. Although the initial purpose of phage therapy was to treat diseases, just like antibiotics, there is a need for future work to include the prophylactic use of lytic bacteriophages to reduce the pathogen load and reduce the risk of infection. However, research is needed to improve phage application under field circumstances (phage composition, application timing, transport, and so on) and to reduce the potential danger factors connected with phage application (dispersal of unwanted genes, effects on fish microbiota). Furthermore, research into naturally occurring phages in the microbiota of cultured animals will reveal their involvement in the organism’s defense against bacterial illnesses and assess the possibilities of using them in a more tailored phage therapy scheme. In addition, intensive viral genome sequencing and investigations for the existence of genetic elements may interfere with bacterial fitness or negatively impact the organism’s health. Such information would also provide us a glimpse into the future of molecularly tailored lytic virions. As a result, more in vitro and in vivo testing is needed before the final release, and any negative effects must be properly documented.

Random genome sequencing, suppression subtractive hybridization, and in vivo-induced antigen technology have all recently been employed to find additional *V*. *anguillarum* virulence-related genes.^[34]^ While these techniques yielded intriguing results, the mechanism involving the pathogenic process is yet to be unraveled. As a result, complementary research methodologies like in vivo expression technology/whole genome sequencing are still being developed. These combined research efforts should reveal how *Vibrio* spp. interacts with its host, leading to the advancement of more efficient illness deterrence and remediation strategies, as well as enhanced detection tools. The latter might be accomplished by incorporating the found virulence markers into existing DNA array platforms for disease identification.

### 4.2. Quorum sensing

Based on bacteria cell-to-cell communication termed QS and aquatic bacterial virulence factors. In this perspective, the V-TG literature evaluates the QS system, a regulatory mechanism that coordinates the expression of certain genes in response to the concentration of signal molecules.^[35]^ It was first reported in the 1970s among 2 luminous marine bacterial species, *Vibrio fischeri* and *Vibrio harveyi*.^[36]^ Among the most studied QS inhibitors are LuxO inhibitors,^[37]^ QStatin [1-(5-bromothiophene-2-sulfonyl)-1H-pyrazole],^[38]^ halogenated furanone and sodium ascorbate.^[39]^ Ever since, studies have shown that the QS system identified in different species of aquatic microorganisms propagates chemical signal molecules^[40]^ that are implicated in the production of virulence factors such as caseinase, gelatinase, lipase, hemolysin, and phospholipase. Similarly, research has focused on QSIs, while others investigate the QS system in controlling the pathogenicity of *Vibrios*, suggesting that interfering with this process would reduce *Vibrios* pathogenicity without producing resistance (Fig. 4B). The bacterium employs a three-channel quorum-sensing mechanism to control the gene expression important for bioluminescence and other features. The 3 channels are fed by the signal molecules HAI-1 (*Harveyi* autoinducer 1), AI-2 (Auto inducer 2), and CAI-1 (Cholera autoinducer 1) (Fig. 4B). QS regulates the expression of different virulence factors in V. *Harveyi*, including an extracellular toxin, metalloprotease, siderophore, type-III secretion system, chitinase, and 3 phospholipase genes. Moreover, QS has been implicated in regulating the virulence of luminescent *Vibrio* in diverse hosts in vivo.^[21]^ Further research into whether signal molecule-degrading bacteria (AI-2 degraders alone or AHL and AI-2 degraders combined, depending on the host system) might impair V. *harveyi* QS, thereby protecting cultured animals against luminous vibriosis, will be intriguing. These microbes could be employed as a new probiotic category if they successfully suppress luminous vibriosis. Given the recent increase in opportunities by exploring the QS system to regulate the expression of virulence toxin by *Vibrio* spp., the results indicate that QSIs can attenuate its QS-mediated virulence and may be a nonantibiotic-founded treatment of fish vibriosis.^[21]^ However, future studies could focus on developing novel, less toxic QSI compounds with explorative mechanism(s) of action for both aquatic and human pathogenic vibriosis.

Meanwhile, QS has gained positive regulating results in producing metalloprotease and extracellular toxins. At the same time, some QSs are negatively regulated while others are independent, as found in aquaculture pathogens (*Aeromonas* spp. and *Edwardsiella* spp.). This could be owing to the expression of distinct virulence factors during different phases of infection. Thus, QS negatively regulated virulence factors for the first phase, and QS positively regulated virulence factors needed at a later stage of infection.

### 4.3. Host immune system

Based on the host immune system and vaccination, the literature points to the immunity and vaccination of fishes, which are constantly threatened by pathogenic microorganisms, especially bacteria.^[19]^ Normally, fish maintain a healthy state of protection against potential pathogens through a multifaceted host defense mechanism. Fish secure protection by continuously renewing skin mucus which has various antibacterial substances like antimicrobial peptides (pleurocidin), proteases (trypsin-like proteases and cathepsin L and B proteases), lectins, and lysozymes. The fish gastrointestinal tract is an antagonistic acid environment containing bile salts and enzymes capable of inactivating and digesting a variety of pathogenic microorganisms. Also, the present activity of antiproteases or factors, as well as iron-binding proteins (transferrin) (lysozymes) in the blood serum have bactericidal activity. Lastly, fishes possess a classical complementary system that supports the pathogen-specific immune response. When activated by interaction with complement components or antibody-antigen complexes (classical complement pathway activation) or by direct contact with Gram-negative bacteria (alternative antibody-dependent complement pathway activation). Both activation routes can cause the death-susceptible pathogen either by creating pores in the bacterial cell membrane or by enhancing phagocytosis via inflammation, lysis, or opsonization Figure S4, Supplemental Digital Content, http://links.lww.com/MD/O432. Pursuing this line of research will become increasingly pertinent in the aquaculture industry. For instance, in live attenuated V. *anguillarum*-vaccinated transcriptome and gene expression analyses revealed alterations in innate and adaptive immunity genes were implicated in the zebrafish model. Suggesting an evoked mucosa immune response in orchestrating the mucosal barricade against *Vibrio*-pathogens via activation of the Th17 pathway.^[23]^ Future research extended to the mucosal immune responses triggered by the immersion route is recommended.

On the other hand, studies on altering immune components and anti-infection at the mucosal entry site will also be inspiring. Nevertheless, investigations on the immune response gene overexpression after vaccination may give useful information on the mechanism of the vaccine against vibriosis during acute phase response in bacterial infection. In addition, investigate the activities that may confer mutational to immune genes during L. *anguillarum* infection and its consequences on fish survival and recovery.

### 4.4. Regulation of toxin functional genes

Based on the regulation of toxin functional genes, V-TG literature investigates the protagonists of the luxO gene both in regulating the production of siderophore and extracellular products in fishes infected with *V*. *alginolyticus* was the cluster focus. Furthermore, null rpoN mutants had significantly increased protease levels and siderophore synthesis but considerably reduced extracellular products hemolytic activity. This suggests an extension of research to understand the mechanism and possibilities of upregulation of siderophore production and downregulation of hemolytic virulence and, in other species, proteins that encode these genes. RpoS is involved in the transcriptional regulation of EmpA^[41]^ and the post-transcriptional regulation of VanT. It stabilizes vanT mRNA at high cell density by repressing the RNA chaperone Hfq, which stabilizes the small quorum regulatory RNAs (Qrr sRNAs).^[42]^ RpoS has a key role in the resistance of bacterial pathogens to heat, as documented by Ma *et al*., 2009.^[43]^ Future research should be strengthened toward understanding the mechanisms that control/regulate RpoS synthesis of extracellular enzymes such as (phospholipases, diastases, lipases, caseinases, hemolysins, catalases, and proteases). Additionally, in Table 3, the future research agenda on *Vibrio* virulence is highlighted and tailored to increase our knowledge of the subject. These include the major areas: identifying important virulence genes implicated in infections of dissimilar hosts; investigating the expression of virulence genes in different hosts during infection (which should provide us with knowledge on the cascade of events during infection); and comprehension of the regulatory mechanisms that control the virulence genes expression (like ToxR regulon, QS, and host signals disturbing virulence gene).

**Table 3:** Thematic and knowledge research streams for future directions

Interconnected thematic	Associated clusters	Research streams for future directives
Pathogenesis and Virulence	Pathogenesis and virulence factors. Characterization and identification of virulence gene. Virulence strains mediated by experimental bacteriophage.	Pleiotropic regulation of pathogenesis. Rapid determinates of *Vibrio* markers for pathogenic and nonpathogenic *Vibrio* spp. Structural elucidation and identification of putative genes for lysogeny
Quorum sensing	Quorum sensing. Aquatic bacterial virulence factors.	Assay of QSI compounds as a candidate treatment in both aquatic and human pathogenic vibriosis research. Mechanisms of extracellular autoinducer in vibriosis pathogenesis. Roles of proteins as regulatory networks of virulence and LuxS quorum sensing system. Analysis of the mechanism to control the expression of diverse *Vibrio* virulence genes and the regulatory role of RpoS in the synthesis of extracellular enzymes.
Host immune system	Immune response genes. Vaccination.	Mechanism of vaccination against vibriosis in the acute phase infection response
Regulation of toxin functional genes	Host-specific invasion factors. Regulation of virulence toxins pathogenesis. Hemolytic phenotypes differential hemolysin gene among marine/aquatic pathogens.	Understanding the mechanism involved in the control/regulatory role of RpoS in the synthesis of extracellular enzymes The differences in hemolytic properties of emerging vibriosis Diversity of pathological changes in septicemia and hemorrhage infections in vibriosis

QSI = Quorum sensing inhibitor.

Source: Author’s own compilation on evaluation summary of Bibliographic coupling analysis and co-citation analysis technique.

## 5. Conclusion

The mapping analysis looked into the nature of the V-TG studies using quantitative and qualitative methods for over two decades. Our finding implies that the V-TG research field is complex, with unclear toxins expression mechanisms that reveal research opportunities. Substantial progress in the V-TG research field was observed by analyzing the obtained dataset using co-citation analysis techniques, and the ultimate research topics were evaluated. On the other hand, bibliographic coupling techniques identify and explore up-to-date emerging themes of attention in the field. By comprehensive content analysis of articles in each cluster, the linkages between the different streams/thematic in the V-TG study paying research streams for better clarification of *Vibrio* pathogenicity presented in graphical abstract. Our findings suggest that *Vibrio* virulence factors are a complex field requiring an interdisciplinary approach consisting of interconnected perspectives such as pathogenesis and virulence, QS, host immune system, and regulation of toxin functional genes, highlighting research agenda that has important consequences for academics and policymakers.

## Author contributions

**Conceptualization:** Hope Onohuean, Uchechukwu U. Nwodo.

**Methodology:** Hope Onohuean, Uchechukwu U. Nwodo.

**Data curation:** Hope Onohuean.

**Formal analysis:** Hope Onohuean.

**Funding acquisition:** Uchechukwu U. Nwodo.

**Validation:** Uchechukwu U. Nwodo.

**Visualization:** Hope Onohuean, Uchechukwu U. Nwodo.

**Project administration:** Uchechukwu U. Nwodo.

**Supervision:** Uchechukwu U. Nwodo.

**Writing – original draft:** Hope Onohuean.

**Writing – review & editing:** Hope Onohuean Uchechukwu U. Nwodo.

## Supplementary Material


