# Supplementary material for: Global systematic mapping of Vibrio species pathogenicity: A PRISMA-guided cluster-based analysis
Source: Medicine (Baltimore). 2025 Feb 28;104(9):e41664. doi: 10.1097/MD.0000000000041664 (PMC11875588; doi:10.1097/MD.0000000000041664)
Supplement: Supplementary file 1 [file medi-104-e41664-s001.pdf]

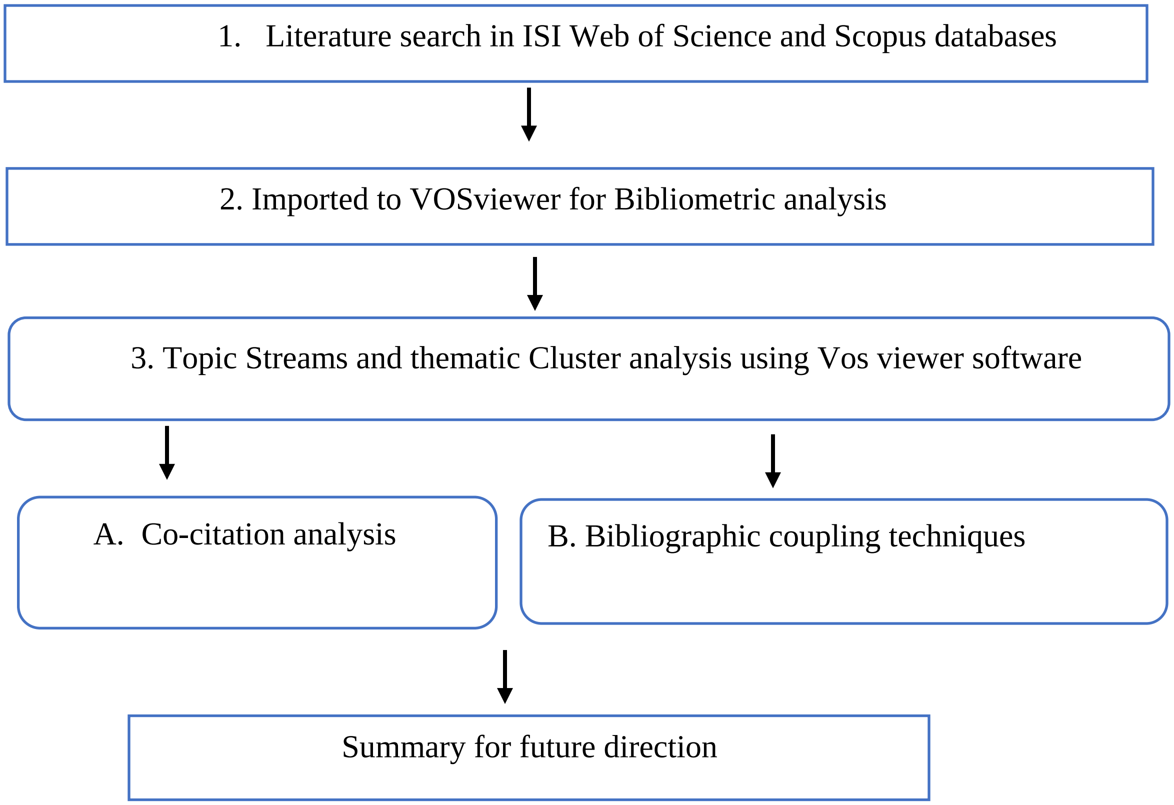

Supplementary Figure 1. Structural Research Method design.

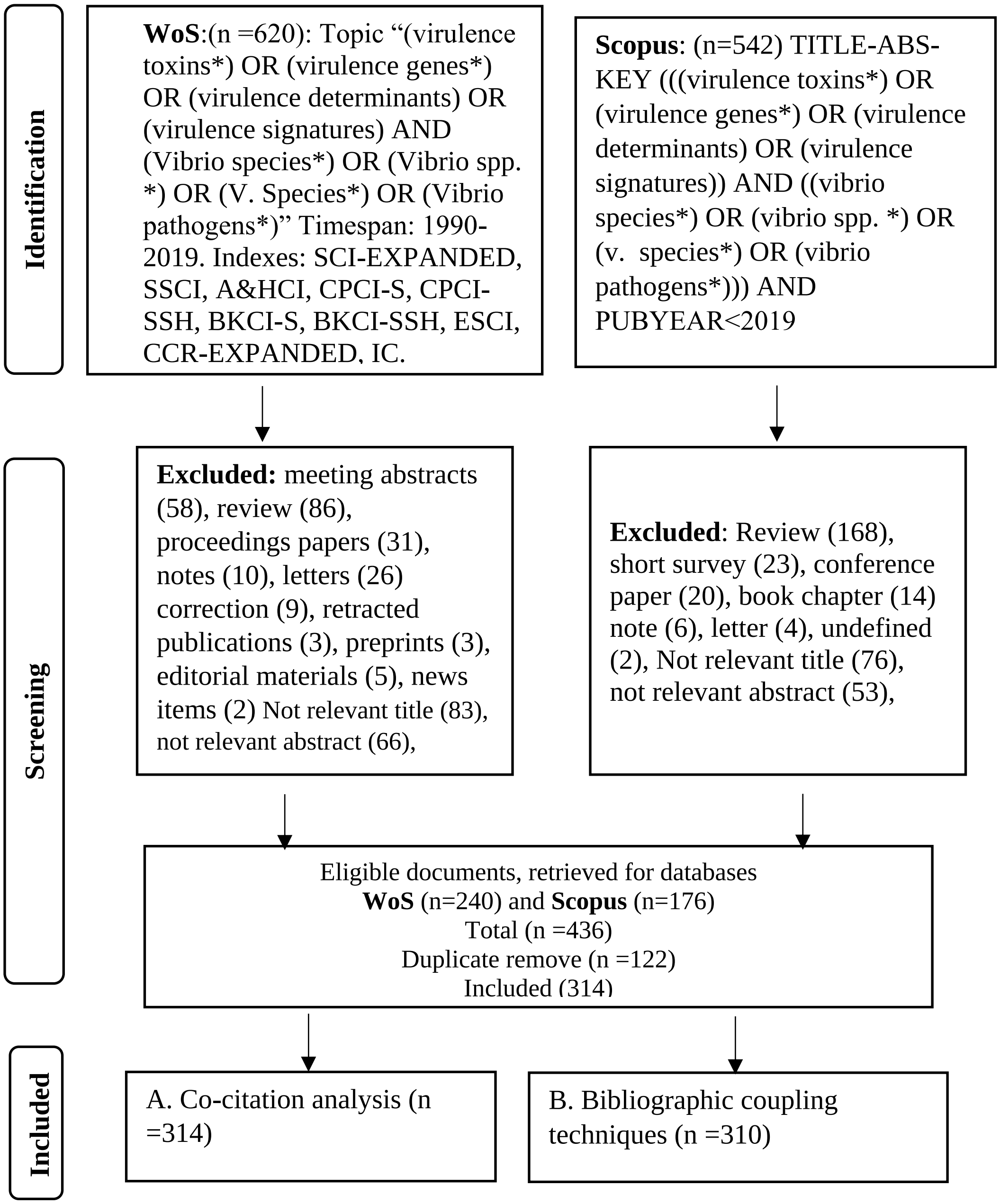

Supplementary Figure 2. PRISMA method for finding, reviewing, and synthesizing papers on V-TG.

c

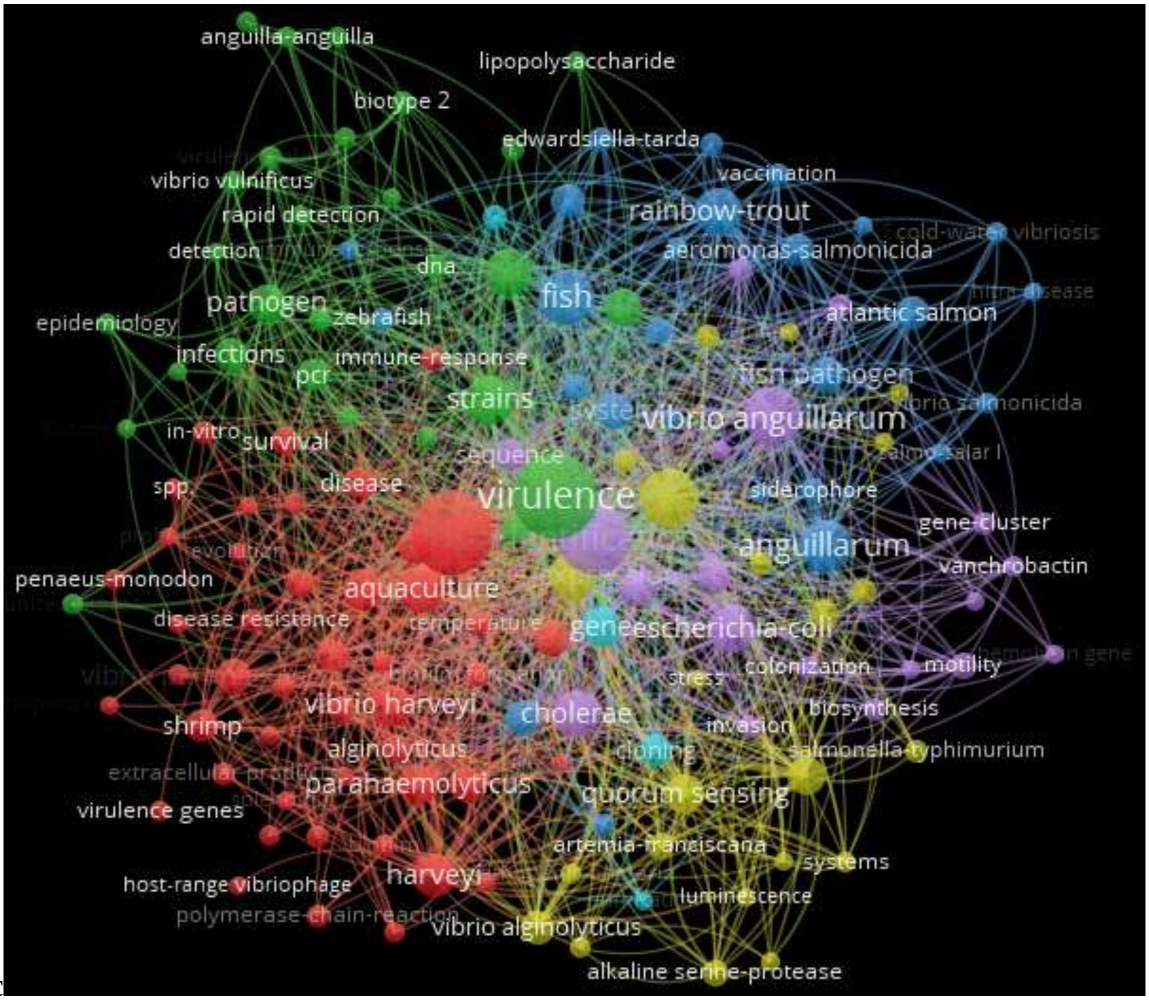

Supplementary Figure 3. visualization of keywords cluster.

Source: VOSviewer co-occurrence analysis technique.

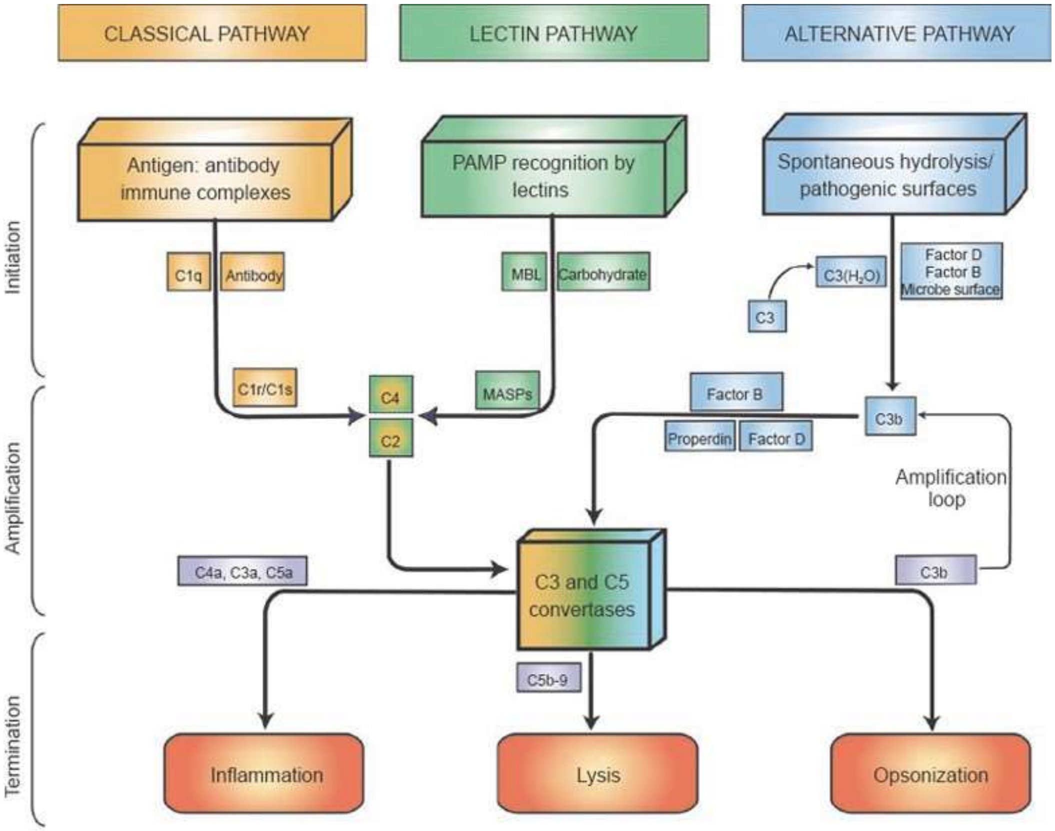

(Dunkelberger and Song, 2010).

Supplementary Figure 4. The link between the classical, lectin, and alternative complement system.

Supplementary Table 1. The distribution of virulence factors linked to pathogenic *Vibrio* infection.

Source: Author's own compilation on the distribution of virulence factors linked to pathogenic *Vibrio* infection.

Species	Toxins factors	In vitro activity	Target system	References
	Cell associated			
V. cholerae , V. vulnificus	Flagellum	Adherence to monolayers	GI tract	[38, 48]
V. cholerae, V. parahaemolyticus	Adhesins	Adherence	GI tract	[5, 9, 49]
V. vulnificus	Serum resistance	Increased growth	Blood	[24, 49]
V. vulnificus	Polysaccharides, acidic	Antiphagocytic, anticomplementary	Blood	[48]
V. cholerae 01, V. cholerae non-01,	Extracellular	FA	GI tract	[50]
V. minicus	Enterotoxin CT			
V. cholerae non-01, V. minicus, V. fluvialis, V. hollisae	Entertoxin LT or ST	FA	GI tract	[51]
V. vulnificus, V. flutract vialis, V. damsela,	Cytolysin LT	Cell lysis, FA, tissue destruction	Wounds, GI	[52]
V. cholerae non-01 V. fluvialis	Cytotoxin LT	Cell death	GI tract	[53]

V. cholerae 01,			
V. cholerae non-01,			
V. parahaemolyticus	Cytotoxin Shiga	Cell death	GI tract
[51]			
V. parahaemolyticus,			
V. hollisae	Hemolysin TDH	Vascular permeability, cell lysis, FA	GI tract
[54]			
V. alginolyticus	Proteases Collagenase	Tissue Destruction	Wounds,
[55]			
V. vulnificus			Cutaneous tissues
V. vulnificus	Protease	Vascular permeability,	Cutaneous lesions
[56]			
	plasma kallikrein-kininactivator		
V. vulnificus	Siderophore	Increased growth, iron	Blood
[57]			
		Acquisition	
V. cholerae 01,			
V. cholerae non-O1,			
V. mimicus, V. fluvialis,			
V. parahaemolyticus	*Mucinase*	Glycoprotein degradation (mucin)	GI tract
[58]			

CT, cholera toxin; FA, fluid accumulations; GI, gastrointestinal; LT, labile toxin; ST, heat-stable toxin; RBC, erythrocytes; TDH, thermostable
